# Considering the Definition of Addiction

**DOI:** 10.3390/ijerph8104025

**Published:** 2011-10-20

**Authors:** Steve Sussman, Alan N. Sussman

**Affiliations:** 1 Departments of Preventive Medicine and Psychology, University of Southern California, Soto Street Building, Room 302, 2001 N. Soto Street, Los Angeles, California 90032, USA; 2 2329 Winthrop Avenue, Apt. 60, Roanoke, Virgina 24015, USA; E-Mail: alansussman67@yahoo.com

**Keywords:** definition, addiction

## Abstract

The definition of addiction is explored. Elements of addiction derived from a literature search that uncovered 52 studies include: (a) engagement in the behavior to achieve appetitive effects, (b) preoccupation with the behavior, (c) temporary satiation, (d) loss of control, and (e) suffering negative consequences. Differences from compulsions are suggested. While there is some debate on what is intended by the elements of addictive behavior, we conclude that these five constituents provide a reasonable understanding of what is intended by the concept. Conceptual challenges for future research are mentioned.

## 1. Introduction

Obtaining a consensual and testable definition of a concept is useful to be able to make inferences regarding how the concept is related to other concepts, and subsequently develop useful applications (e.g., policy, services offered [1]). Unfortunately “addiction” is a concept that has been the subject of much debate [2–9]. Delineation of its definitional elements may be a step toward eventually achieving consensus and operationalization of this construct. At its origin, “addiction” simply referred to “giving over” or being “highly devoted” to a person or activity [10], or engaging in a behavior habitually [11], which could have positive or negative implications. Over the last 400 years, some statements made about addiction began to frame it as involving strong, overpowering urges and, over the last 200 years this word has become considered more and more disease-like in connotation [12]. Many conceptualizations of the addictions pertain to imbalance of the central nervous system in some way, and these conceptualizations date back to the end of the 1700s [13]. It is more recently used as a concept having neurobiological underpinnings [14–19]. Descriptions of addiction that map onto measurable criteria could serve as phenotypes that might maximally explain gene-environment interactions in this arena [20], and best serve prevention and control efforts.

### Literature Search

As a start to grappling with views on definitions of addiction, we engaged in an electronic search of Google Scholar, pairing the term “addiction” with “definition” (on April 7, 2011). The first 500 of 172,000 web pages was examined, revealing 40 relevant citations. We also engaged in the same search on OvidMEDLINE (1948-June Week 4, 2011; on July 3, 2011), revealing six citations but no new ones. Finally, we engaged in a search of PsycINFO (July 23, 2011). Simply pairing the terms revealed 2,994 peer reviewed articles. However, only two of the first 100 provided relevant information (*i.e.*, pertaining to definitions of addiction). Thus, a second search using the phrase “definition of addiction” was conducted, revealing 47 citations total and 33 peer-reviewed articles. Of these, two citations were unique and relevant to grappling with the definition of addiction. All articles found through this search, along with others obtained through previous work and word of mouth, are included in this review (total = 52 published sources that discuss the definition of addiction).

To our knowledge, this is the first systematic electronic literature review of the concept of addiction. Based on this literature review, it appears that addiction is conceptualized as being composed of multiple elements [6,21]. We highlight explicitly stated elements common across at least 20% of the 52 papers that address the definition of the addictions. However, a quantitative analysis was not provided here that examines number and type of element referred to as a function of study characteristics. Rather we took a qualitative approach that summarizes the most commonly agreed on elements of addiction and discusses philosophical concerns regarding necessary and sufficient conditions for a psychological state or pattern of behavior to *be* an addiction. It is true that over 75% (ns > 39) of the papers in the set do mention all of the criteria except for satiation as being elements of addiction. Only 20% (n = 10) of the 52 studies uncovered mentioned satiation as a definitional element of addiction; however, we view it as an essential element to the concept and one which has obvious treatment implications. While we felt that a quantitative analysis would not be of much incremental benefit, others may disagree. These five elements, which have been most popularly suggested as being constituents of addiction, are discussed next.

## 2. Results

### 2.1. Feeling Different

In most cases, an addiction does not develop overnight. In general, when contemplating addiction, one often thinks of it in terms of a process. Upon the initialization of the “addictive process” [4,22,23], one pursues some course of action for appetitive effects or motives (e.g., pain reduction, affect enhancement, arousal manipulation, or fantasy). Different addictive behaviors have been empirically clustered as serving hedonistic (e.g., drug use, sex, gambling) or nurturant (e.g. compulsive helping, work addiction, shopping addiction, love, exercise) motives [24,25]. However, other or additional motives are plausible (e.g., to achieve fantasy or oblivion [3]), and all addictions may share in common a function to shift subjective experience of self [26].

The addiction process unfolds for some individuals but not others, and may reflect individual differences prior to engaging in the addictive behavior or as the individual continues to engage in the addictive behavior (*i.e.*, individuals may vary along a dimension of “addiction proneness”). Anecdotally, many self-described addicts have reported feeling “different” from others long before developing readily identifiable addictions. This includes feeling relatively uncomfortable, lonely, restless, or incomplete [23]. Once a behavior is tried that decreases or eliminates the baseline sense of discomfort a process begins to unfold. It is possible that 50% of the variance of addictive behavior is attributed to a genetic cause of this subjective sense of discomfort [18,27]. The extent to which there exist persons in-born for addiction remains a subject of debate [26].

Alternatively, many persons report not feeling different prior to engaging in a problematic addictive behavior. Among these individuals, a behavior may be tried that is perceived as highly valued or enjoyable, possibly with effects that occur rather rapidly, that one desires to repeat [28]. In this instance, a process begins to unfold inducing a contrast between an enhanced or potentially addictive behavior-induced state of arousal, affect or cognition, and a baseline state of arousal, affect, or cognition. The initial reaction to the potentially addictive behavior may be experienced as more positive than with other persons (among those relatively prone [8]). That is, addictive “appetites” may fall along a continuum [2], and those persons at one extreme may find certain behaviors particularly enticing. Involvement in extreme levels (frequency or valence) of these behaviors, which tend to be subject to social or other consequential restraints, may identify addictive levels of behaviors [12].

### 2.2. Preoccupation with the Behavior

A second aspect of addiction considers excessive thoughts about and desire to perform a behavior, excessive time spent to plan and engage in the behavior, and possibly recover from its effects (e.g., from “hangovers”), and less time spent on other activities [29], despite potentially diminishing appetitive effects [30,31]. That is, the addictive behavior “spills over” into several dimensions of one’s daily life. This may be labeled more generally as “preoccupation.” For example, a two-pack-a-day cigarette smoker may report often thinking about smoking cigarettes (particularly when restricted from smoking, or at certain points during the day when one is most likely to smoke), or thinking about anti-smoking control efforts, may invest a great deal of money to continue to purchase cigarettes, may have a cigarette in hand 280 minutes per day (approximately one-third of the waking day; a behavioral aspect of preoccupation), and may report experiencing discomfort upon cessation of smoking for more than a couple of hours.

Interestingly, it is not known to what extent addictive desires operate on neurobiological processes differently from regular desires [2,3]. However, addictive behavior-induced repetitive firing of certain brain systems (e.g., mesolimbic dopamine) does result in brain adaptations (e.g., activation of glutamatergic system; decrease in production of mesolimbic dopamine), suggestive of a “hijacking” of the brain due to engagement in any of a variety of substance or process addictive behaviors [32–34].

Tolerance and withdrawal are the two hallmark criteria of physiological addiction, and, arguably, may also be considered as aspects of a more general concept of preoccupation (or as features that contribute to preoccupation). Tolerance refers to the need to engage in the behavior at a relatively greater level than in the past to achieve previous levels of appetitive effects. As tolerance increases, one likely spends more time locating and engaging in an addiction. Thus, tolerance may indicate increasing preoccupation. Withdrawal refers to physiological or acquired discomfort experienced upon abrupt termination of an addictive behavior. If withdrawal symptoms exist, and worsen, one is likely to be spending more and more time recovering from the after-effects of the addiction, and focused in thought and action on how to cope (e.g., by using again). That is, one is more preoccupied with the addiction when one is spending more time locating, engaging, and recovering from that behavior, and this may reflect processes of tolerance and withdrawal [35–37].

Possibly, related to tolerance or withdrawal, is the notion of craving. Craving or urges to engage in a target addictive behavior has been a hallmark defining feature of the addictions for a long time [13,38]. Craving is not necessarily the same thing as physiological withdrawal and may, in fact, be more central to the concept of addictions [34]. For example, several highly addictive drugs (e.g., cocaine) are thought to not have strong physiological withdrawal symptoms (e.g., such as “the shakes” as with alcohol); rather, they are identified by drug seeking behavior while in the midst of accumulative negative consequences [34]. Some addicts who are new in recovery may even maintain a subjective sense of fear that catastrophic events will occur if they continue to refrain from their addictions [23]. Craving has been proposed as a diagnostic feature of the addictions to be added to the DSM-V [39]. While there is some ambiguity regarding the definition of craving (e.g., this concept may overlap with implicit expectancy cognitive processes [19]), at least *prima facie*, the definition appears to refer to an “intense desire” to engage in a specific act [3,40], that reoccurs and about which one often conforms [40].

### 2.3. Temporary Satiation

A third element of the concept of addiction is “satiation”. After acute engagement in an addictive behavior, some period of time may occur in which urges are not operative, addiction craving is “shut down”, only to return soon [3,12,38]. This satiation period is not well studied or considered. Some thoughts regarding this period pertain to a sense of distraction from life problems or feeling temporarily self-sufficient or nurtured [41,42]. If these feelings continued, arguably, one may speculate that the individual would have achieved a resolution of the subjective sense of discomfort (or “disbalance”) that precedes engagement in the addictive behavior [43].

Satiation may be examined from the perspective of the Incentive-Motivational Model [12,44,45], which examines in part how an addictive behavior may elicit satiation of emotional expectations through its incentive value (e.g., feeling “incentivized”). From this perspective, non-addictive alternatives over time may lose incentive value. That is, even though the addiction may not achieve satiety as well as it used to, its relative incentive value compared to non-addictive alternatives may increase [45]. That is, while there may be some discomfort in trying to achieve satiation, alternatives may provide even less of a chance of achieving a sense of satiation. Therefore, an iterative pattern continues involving a period of participation in an addictive behavior followed by a period of satiation.

One other notion possibly pertaining to satiation (as well as to loss of control, discussed below) is that of psychological reversals [46,47]. This notion is the idea that people may fluctuate sharply between two or more experiential states (e.g., shifting back and forth between experiencing a sensation seeking state, to a goal-oriented/calm state). Biological needs, valence *versus* time delay of addictive behavior-related rewards, and a feeling a sense of frustration *versus* satiation, can shift one from one state to another [46,48]. That is, when frustrated the individual may seek out an addictive behavior, and when satiated the individual may temporarily avoid such temptations. The shift that may occur (e.g., Dr. Jekyl to Mr. Hyde) appears to reflect a lack of control, though behavior may appear well-controlled within each mood state.

Arguably, there may be instances in which a person suffering from an addiction reports no longer being able to achieve satiation. If so, some researchers might suggest that satiation should not be considered a defining element of addiction. It might rather be considered as a construct which interacts with addiction [49]. Alternatively, it is feasible that satiation almost always is achieved even if for brief periods, possibly directly following the first moments of onset of an addictive behavior, and when not satiated, satiation is still sought.

### 2.4. Loss of Control

Among the defining elements of addiction, loss of control has a rather long history [12,13]. One may report desiring to stop an addictive behavior but, even so, not having the ability to precisely predict when a bout with the behavior will be initiated, how it will be manifested, or when it will stop. That is, the addictive behavior may become increasingly more automatic [4,12,15,19,50–54]. Difficulty in refraining from an addictive behavior despite attempting to do so may be central to a loss of control aspect of addiction (see Heather [19] on “akrasia”). Many persons claim to be struggling with an addiction; feeling compelled, sensing incomplete control; and it is observed that they may disregard even basic self-care, suggestive of a loss of will [8].

Incomplete memory access appears to be a common feature of addictions. According to Campbell [29], the “cognitive impairment” associated with an addiction emerges only when a specific addiction associated with harmful consequences produces a simultaneous positive emotional response. This attentional narrowing minimizes or negates the memory of the negative effects or consequences of previous addictive behavior experiences (or access to aversive memory). Phenomenologically, due to these memory effects, recovering addicts with some “sober time” may look back at their using days as being disordered, illogical, fragmented, destructive, and nonsensical [41].

Impulsiveness is another descriptor that has been used to indicate addiction-related loss of control [55]. This aspect of the addictive process might be identified as including spontaneous urges to engage in the addictive behavior about which executive inhibitory processes fail to operate due to actions of addiction-related reinforcers on separate memory systems (implicit *versus* declarative systems [56]). Possibly, the existence of separate memory systems may account for both “incomplete memory” and “impulsiveness” descriptions of the loss of control aspect of addiction [51]. That is, the implicit system may facilitate attentional narrowing and impulsive behavior associated with previously reinforcing addiction-related events that are strongly embedded in memory, whereas the declarative system may fail to be operative in “trigger” (high risk) situations, to inhibit a relatively automatic (self-destructive) chain of behavior [51,56].

On the other hand, executive planning (declarative cognitive) processes are involved in the addiction seeking process (e.g., one may need to be innovative to acquire their drug of choice [2,43]), many people appear to mature out of addictions [2], and many times if sufficient justification is provided a person may stop engagement in an addictive behavior [2]. Such examples could suggest that persons suffering from addictions simply are making choices for pleasure in accordance with their lifestyle preferences (and they may have very strong desires for pleasure), contrary to normative expectations [2]. That is, people may have very strong, regular, appetitive desires that outweigh other alternatives and cause negative consequences; but are engaged in not due to an obvious lack of behavioral control *per se*. The relative emphasis placed on appetitive *versus* loss of control aspects of addiction apparently vary as a function of age; adults tend to view the loss of control aspect as more important than do adolescents [36].

### 2.5. Negative Consequences

A fifth defining element of the concept of addiction is the existence of negative consequences. In general, at some point, negative consequences tend to ensue due to engaging in an addictive behavior (e.g., physical discomfort, social disapproval, financial loss, or decreased self-esteem [57]). Continuing to engage in the addictive behavior after suffering numerous negative consequences often has been a criterion of dependence on the addictive behavior [4,29]. Stopping the behavior becomes difficult for several reasons, including influence of the cognitive salience of immediate gratification resulting from the addictive behavior (*i.e.*, satiation) relative to its delayed adverse effects. The individual also may fear having to cope with day-to-day perceived stress and other life experiences upon cessation (possibly due to accumulation of addiction-related consequences, or having to endure “raw” emotional experiences without concurrent self-medication, having failed to learn to cope without use of the addiction [23]), as well as suffer withdrawal-related phenomena [22,58,59]. Thus, the addiction persists, incurring negative effects while also providing maintenance functions.

Negative consequences may vary across contexts. For example, arrests for drinking and driving may not be well-enforced in some countries (e.g., some rural areas in Southeast Asia), or may be enforced very strictly in other countries (e.g., Sweden). Thus, the legal consequences related to drinking alcohol may vary across contexts. Physical consequences may vary likewise (e.g., there may be fewer injuries and deaths related to drinking and driving in locations where drinking-diving laws are well-enforced [27]). Also, the social consequences of alcohol use and other addictions vary across history [11] and cultures [60], for example, due to differential tolerance of public display of drunken (or high) behavior. Possibly, role consequences (e.g., difficulty fulfilling one’s role as parent, spouse, or coworker) may be a relatively invariant aspect of negative consequences that operates across different addictions [27]. That is, if a person is unable to perform their societally-defined roles in the world (which sets the parameters of their overall contribution to self and others) due to their problematic behavior, they are maximally likely to be labeled as “addicted”.

### 2.6. Differentiating Addiction from Compulsion

Some people view non-drug use addictive behaviors, such as pathological gambling or shopping, as being “compulsions” [41], that involve (a) spontaneous desires to act a particular way, (b) a subjective sense of feeling temporarily out of control, (c) psychological conflict pertaining to the imprudent behavior, (d) “settling for less” to achieve the same ends, and (e) a disregard for negative consequences. Others use the term “compulsion” more narrowly. Some may define this term as a simple but intense urge to do something; only one aspect of addictions but centrally definitive of obsessive-compulsive disorders [61]. It may be defined even more precisely as an intense egodystonic (separate from self) urge to engage in a simple, repetitive activity, to remove anxiety [55]. Such activities may include repeated washing of hands, tying of shoes, or bathing, or restricting areas in which one will travel (e.g., not walking on cracks). A narrow definition of compulsion does not, primarily, consider the interplay of higher-order cognitive processes, such as the planning that may go into completion of a cycle of addictive behavior. (Arguably, however, someone may decide to wash their hands where there is plenty of soap available and the facility is considered very clean; this may involve planning). Also, the act may accomplish a temporary removal of anxiety, but it tends not to be experienced as pleasurable at any point in the engagement of the behavior [61]. Conversely, an addiction, by definition, involves the attempt to achieve some appetitive effect and satiation through engagement in some behavior. In fact, a whole constellation of purposeful behavior may be involved in attempts to achieve satiation [60].

## 3. Discussion and Conclusion

A series of complex, associated behaviors may be engaged in to continue to achieve appetitive effects. The problem with continued engagement in addictive-related behaviors is that over time they lead to negative side effects. The “addict” may then try to figure out new behaviors to achieve similar appetitive effects, while trying to avoid negative results. Over time, negative consequences may be greater than the positive consequences of engaging in any number of addictive behaviors. However, the participant may continue to engage in the behavior for several reasons. These reasons may include considering the behavior as a compromise between aspects of daily experience about which the participant feels a lack of control or accomplishment and aspects of experience the participant can manipulate. The behavior may be of a sort the participant can engage in relatively easily, and may still serve as a “short cut” to obtaining affective goals. The behavior may become a lifestyle, a means of existence. The stance the participant may take depends in part on the other activities about which the participant has access, or involvement. In the midst of the engagement in the addictive behavior, other competing behaviors may or may not be of interest unless woven into the fabric of the addictive behavior [45].

Formalized treatment may become imperative. Several overarching models have been proposed, contingent on whether or not the individual is considered to be held responsible for acquiring (yes/no) or resolving (yes/no) the addiction problem. Marlatt [57] categorized the acquiring-resolving typology as moral (where the individual is held personally responsible for both; considered weak-willed), medical/disease (responsible for neither; being physiologically disordered; must rely on external support for prevention of addiction and to achieve and maintain sobriety, if addiction has set in), enlightenment (responsible only for the development of the problem; the person made repeated mistakes, leading to the problem which demands external support), and compensatory (being vulnerable, and responsible only for the solution; must take responsibility for change). Of course, individuals may vary along quantitative dimensions of voluntariness regarding acquiring and resolving an addictive disorder. Placing persons at different points along two such dimensions may be a more accurate portrayal of individual differences. More thinking on how conceptualizations of addiction interface with treatment implications is needed.

### 3.1. Philosophical Concerns

Since this is an inquiry into the content of a concept, or perhaps into the essence of the phenomenon denoted by that concept, a glance at some of the philosophical concerns this inquiry presents may be in order [1]. A natural first step is to look for the necessary and sufficient conditions for a psychological state or pattern of behavior to *be* an addiction. But it must be emphasized that there may be no such necessary and sufficient conditions for defining the term “addiction” or for being an addiction. Nevertheless, when we speak of “addictions” we are speaking of something rather than nothing and an inquiry into what it is may be illuminating.

It can be misleading to say we want to *define* “addiction”; that is, this may appear to say that our interest is in lexicography, in the *word* “addiction”. But, of course, we all know how to use the term; we do not have to run for a dictionary whenever one speaks of addiction. Rather, our concern is to say what addiction *is*. Ideally, we would like to discover the necessary and sufficient conditions for someone to have an addiction, and to do so in such a way as to provide real illumination about the sort of phenomena we have in mind when thinking about addiction. We *know* we are talking about *something* that we recognize in certain clear cases and we want to know just what it is. Of course, we must be careful. There was a time when our ancestors wanted illuminating definitions of the ether that filled space, the spheres in which heavenly bodies were embedded as they spun around the earth, and the caloric that brought heat and left a burning object as it reduced to ash. As it happens, while our aforesaid ancestors were talking about *something* that they clearly did observe, the ether, spheres, and caloric did not exist. Natural philosophers may have tried mightily to define these “phenomena” in scientifically illuminating ways, but their efforts were hopeless for, again, these “things” did not exist. As we learn about the world we experience we do not merely learn more about things we know exist; we also change our beliefs about what things exist, even things as obvious as caloric and the ether [62].

Our “ontology” is the set of (kinds of) things that must exist if our beliefs are true. The point to note is that scientific progress involves not just changes in beliefs about the properties of and relations between the entities in our ontology—the ontology itself may well have to change. One often enlightening fact, or even guide, about those elements of our ontology that are destined for elimination is that efforts to provide illuminating necessary and sufficient conditions for their occurrence are frustrated. It is possible that, at some point in the evolution of our scientific arena, “addiction” *per se* may have to be eliminated from our scientific ontology. Of course, it would not follow that when we speak of addiction, we are speaking of *nothing*, just that we do not yet know *what* we really are talking about. We don’t even know if there is one natural phenomenon involved [62,63].

Well, if addiction does not exist, what *are* we talking about when we use the word “addiction”? First, we may be talking about various things that can occur separately. For example, if we administer a lot of morphine over some time to an individual, he will become subject to a nasty withdrawal if we suddenly stop administering it (an aspect of preoccupation). He might also want us to stop the morphine immediately and go “cold turkey”, to get the “monkey off his back”. In these cases it is common to say that the individual is “addicted”, although he did nothing to bring it about and does not want it to continue (no appetitive effect). Also, quitting may not be particularly difficult (no or little loss of control). On the other hand, some researchers would define addiction, in part, as a behavioral pattern that the individual’s choices led to [2], which now has led to a personal sense of loss of control [8]. Moreover, some others would speak of addictions in which there is no real withdrawal syndrome at all (but there would be another manifestation of preoccupation)—a relatively new usage [33]. Very recently the emphasis has changed from addicting substances to various other addictive behaviors [59,60]. There may be a tendency to group all such instances together when in reality they may be different entities. Thus, possibly, the five definitional elements presented in this paper may represent different phenomena—they may not, only in conjunction, lead to a whole concept that we term “addiction”.

Conversely, perhaps addiction is a disjunctive concept; that is, its definitional elements serve to create the concept. That is, perhaps one is addicted if he or she is demonstrates withdrawal-like phenomena or other aspects of preoccupation with an addictive behavior, even if he or she fails to demonstrate other definitional elements, such as loss of control. Certainly, the way addiction is used in everyday language is diverse. Its use in science may be less so, but still may refer to one definitional element *or* another, rather than one definitional element *and* another.

Thus, there are several possibilities regarding what the relationship is between “addiction” and the five aforementioned definitional elements. We note at least four. First, each of the elements alone, or in any combination with one or more of the others—perhaps all—may be a necessary condition of addiction. Second, each of the elements alone, or in any combination with one or more of the others— perhaps all—may be a sufficient condition of addiction. Third, the five criteria elements may provide a family resemblance concept of the phenomenon or phenomena denoted by “addiction” [64]. A family resemblance is best exemplified by, well, a family. We may say that all of the Smith's appear similar (e.g., on any number of elements contained within the family set, such as weight, eye color, hair, last name) even though no two are identical in descriptors (elements) examined and two of them within the group (set) may have no feature in common. Say Al and Mary have no one feature in common, and yet somehow Al and Mary both “look like members of the Smith family” (e.g., Al may have Smith weight and hair, but not eye color and last name; whereas Mary may have Smith eye color and last name, but not weight and hair). If there are two people who are labeled as addicted, but share none of the same elements in common (e.g., Al may experience loss of control and negative consequences whereas Mary may experience appetitive effects and preoccupation), by the way, we can expect to discover new phenomena of addiction that bear only a family resemblance to the ones we now recognize [64]. There is a fourth possibility; the definitional elements may turn out to be a grab bag of notions that fails to name any real kind of thing, any part of “the furniture of the universe”.

A deeper understanding of the relevant phenomena may well lead us to see the *essence* of addiction as a certain brain state—say a particular degradation of the pleasure system—however that state came about [18]. As an analogy, consider “water”. Once chemistry did its job, we came to use “water” to denote a certain natural kind, H_2_O [63]. We now say that in the old days folks were often mistaken when they called certain liquids “water”—even many of the constituents of the Chicago River. In summary, of course, there are phenomena of interest and importance at issue here, but the effort to define addiction in a way so as to make it a respectable scientific term is, at present, likely to commit errors of inclusion or exclusion.

There is another respect in which the concept of addiction may fall short of scientific respectability. Often, at least, one does not call something an addiction unless the relevant behavior is considered in some way “bad”. That is, typically, for example, one does not claim to be addicted to breathing (c.f., [38]). Of course, there is also talk of “positive” addictions, such as work or exercise, but generally such talk is employed to show how these behaviors can lead to (the definitional element of) negative consequences [58,59], along with associated “bad” connotations (e.g., the person being selfish or out of control (a “loose cannon”)). There is yet another related problem with the notion of addiction. The concept of an addiction is inextricably embedded in an interconnected matrix of common sense concepts such as desire, will, compulsion, pleasure and more. None of these concepts have the kind of clarity we want from terms in a well developed theory, terms such as H_2_O, electron, glutamate or dendrite [62].

### 3.2. Limitations and Conclusions

From this discussion, and additional thought, one may infer at least four limitations with the presentation of the definitional elements provided in this paper. First, there was no attempt made in this paper to discuss how to measure each definitional element. For example, any of the definitional elements might be measured through use of self-report, implicit cognitive techniques, or through participant observation [27,51]. Rather, the goal was more modest, and future empirical work is needed (though some direction exists [58]).

Second, and a related point, is that it is not clear to what extreme a behavior must fall before it would be considered an “addiction”. That is, one may ponder whether or not merely occasional (e.g., a couple of gambling sprees) or constant engagement (e.g., gambling to the point of bankruptcy) in an addictive behavior could indicate appetitive motive, preoccupation, loss of control, temporary satiation, and occurrence of negative consequences of sufficient magnitude or frequency to label the behavior as an addiction.

Third, there was no discussion of how the five elements might be related to each other. (This assumes that all five elements are necessary and sufficient constituents of addiction.) The reader may infer that we intended the operation of a linear process (appetitive motives to preoccupation to temporary satiation to loss of control to negative consequences). However, that was not intended. It is possible for example, that several feedback loops may be operative (e.g., appetitive engagement to temporary satiation to preoccupation to appetitive engagement again, eventually leading to loss of control and negative consequences). It is also possible that each element impacts in some way all other elements, a rather complex process.

A final limitation is that we did not place emphasis on the knowledge that addiction may be context-dependent. That is, what is considered an addiction in one social-environmental location may not be considered addiction in another location [27]. For example, daily use of marijuana may be considered an addiction in the United States but not in some settings in Jamaica. Likewise, at a more specific level of inquiry, the five definitional elements offered herein (appetitive engagement, preoccupation, satiation, loss of control, and negative consequences) may vary in application in different social-environmental locations. It is likely that more extreme levels of each of these elements would be considered definitive of addiction when examining marijuana use in settings in Jamaica in comparison to the US; that is, it may be difficult to remove addiction and its defining elements from varying normative standards of behavior. A common scenario is one in which a person forgets where they parked their car after a drinking episode. This scenario might be interpreted as a negative consequence of drinking, or as a humorous story, depending on the social norms in operation when the event is being vocalized (e.g., at church *versus* a college fraternity gathering).

Of course, if there were no negative consequences, and if the experience was always fulfilling, one may speculate that the addictive behavior would continue, and may or may not even be considered addictive (c.f., or as a positive addiction). However, continued engagement in the addictive experience may limit the options to engage in alternative experiences. While not leading to dramatic negative consequences *per se*, the experience may conflict with other desired patterns of behavior. Arguably, most addictive behaviors do not simply terminate when one desires to stop. There is a “recovery period” in which one anticipates feeling better physically, financially, or otherwise. Then one may consider other pathways of action. These other pathways may be harder to achieve the more entrenched one has become in the addictive behavior. In fact, involvement in addictive behavior may reflect a dialectic; that is, an approach-avoidance response. That is, the participant desires the behavior, its satiation, but also desires its avoidance; one equivocates in preference. This equivocation may serve as the experiential substrate of an appetitive motive and satiation, and loss of control and preoccupation, and may itself be experienced as a negative consequence.

It is hoped that the five definitional elements of the concept of addiction presented herein provide a heuristic basis for greater consensus and forward thinking. Certainly, to define addiction in a meaningful, important way (*i.e.*, not mere lexicography) it is necessary to develop a well confirmed, substantive *theory*—just as the definition of water requires chemical theory. Definition, theory construction and confirmation are not separate activities. And it is quite possible that there is no theory that could justify one precise concept of addiction that would cover all the phenomena of concern, including encompassment of all five definitional elements. Or not—it depends on further empirical and theoretical work. Much work remains to be done to fine-tune the definition of addiction, which will entail theoretical modeling.
